# Acute bilateral anterior circulation stroke due to anomalous cerebral vasculature: a case report

**DOI:** 10.1186/1752-1947-2-188

**Published:** 2008-06-02

**Authors:** Brian F Menezes, Beverley Cheserem, Jothy Kandasamy, Donnacha O'Brien

**Affiliations:** 1 The Walton Centre for Neurology and Neurosurgery, Liverpool L9 7LJ, UK; 2 Department of Medicine, University Hospital Aintree, Liverpool L9 7AL, UK

## Abstract

**Introduction:**

Simultaneous bilateral cerebrovascular infarction is relatively rare and its initial presentation as a space-occupying lesion is extremely uncommon. However, bilateral infarction can result from unilateral occlusion of anomalous cerebral vasculature.

**Case presentation:**

We report the case of a man presenting with lower limb weakness and aphasia of acute onset with initial computerised tomography suggesting bifrontal neoplasm. However, further investigation confirmed bilateral anterior cerebral artery territory infarction with a hypoplastic left anterior cerebral artery with the right anterior cerebral artery supplying both frontal lobes (an anatomical variant). We present the clinical and diagnostic features of this presentation and attempt to ascertain, by reviewing existent medical literature, the frequency and patterns of structural variations in cerebral vasculature.

**Conclusion:**

Simultaneous bilateral cerebral infarction can be the result of a unilateral cerebral artery occlusion and this can potentially mimic a space-occupying lesion. Anomalies of cerebral vasculature are not as rare as is usually believed and this should be borne in mind when investigating unusual presentations of cerebrovascular infarction.

## Introduction

Cerebrovascular infarction is a well-recognised clinical disorder but simultaneous bilateral infarction is relatively rare and its initial presentation as a space-occupying lesion is extremely uncommon.

Several morphological variations of the circle of Willis exist. In the Hodes et al. autopsy series, only 18% of specimens of the circle of Willis were found to be anatomically normal [1] and Krabbe-Hartkamp et al. reported that 42% of subjects showed a complete circle of Willis on magnetic resonance angiography [2]. Maurer et al. reported that an anaplastic A1 segment was found in 0.3% to 2.0% of individuals [3].

Given this, bilateral cerebral infarction may be the result of unilateral cerebral artery occlusion and this can account for its unusual initial presentation as an intracranial tumour.

## Case presentation

**Figure 1:**
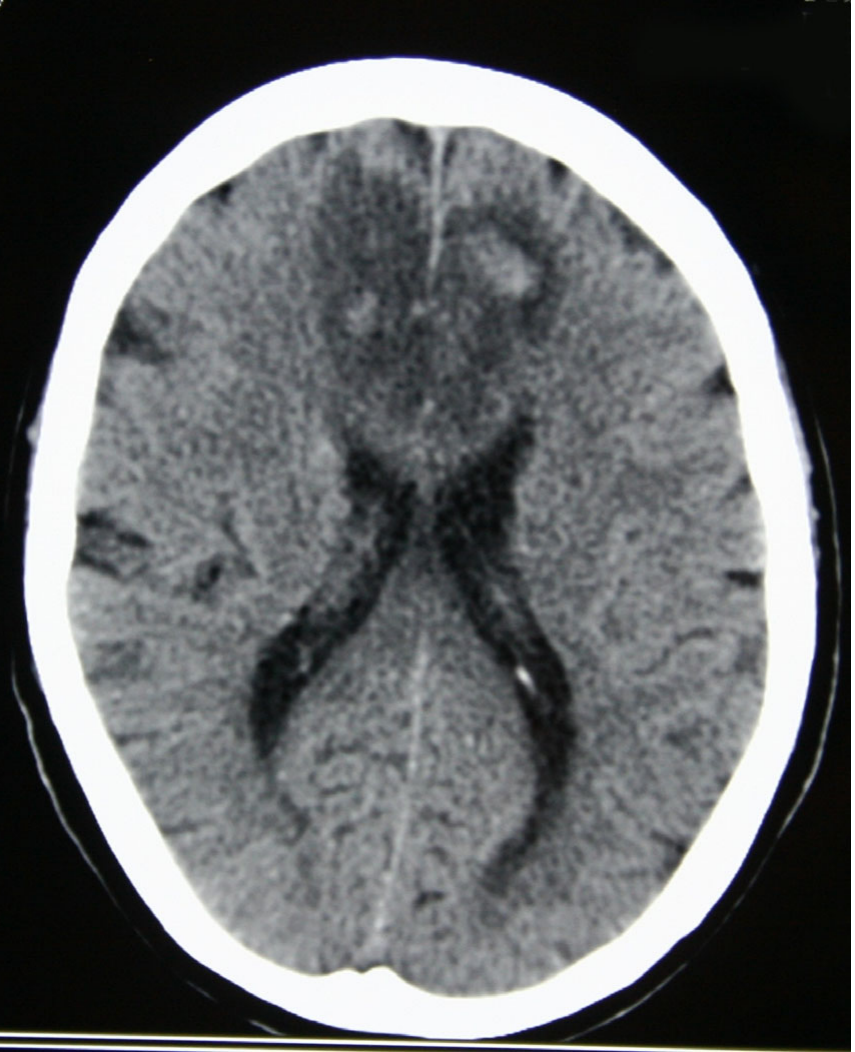
Computerised tomography of the head demonstrating a bifrontal area of hypodensity reported as a parasagittal space-occupying lesion.

A 71-year-old man was admitted to his local hospital with sudden onset aphasia and weakness in his lower limbs, which had occurred whilst playing golf. He reported no loss of consciousness, headache, nausea and/or vomiting, convulsions or incontinence. He had no significant past medical history and had no vascular risk factors. Routine full blood count and biochemical analysis were normal. Cranial computerised tomography (CT) and magnetic resonance imaging (MRI) performed in the original referring hospital were reported as demonstrating a bifrontal parasagittal space-occupying lesion crossing the head of the corpus callosum (thought to be a primary cerebral tumour); see Figure 1. He was therefore transferred to the regional neurosurgical unit for further management.

**Figure 2:**
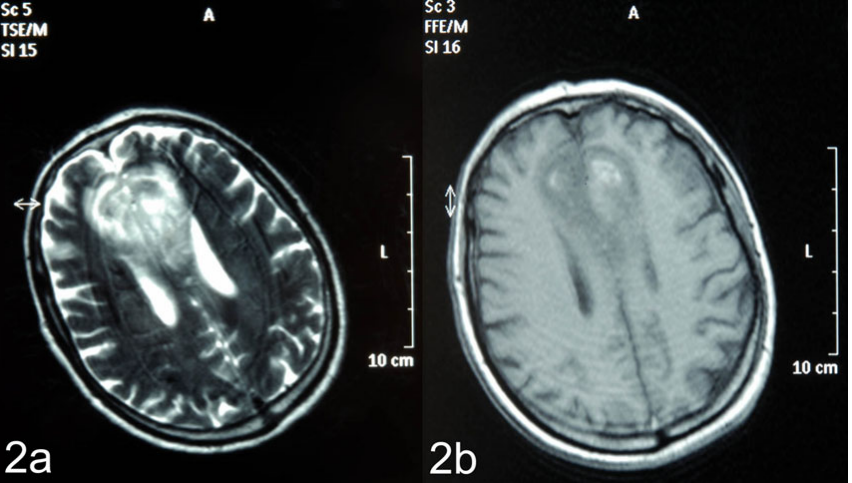
T1/T2 weighted magnetic resonance images initially erroneously reported as demonstrating a bifrontal neoplastic space-occupying lesion.

**Figure 3:**
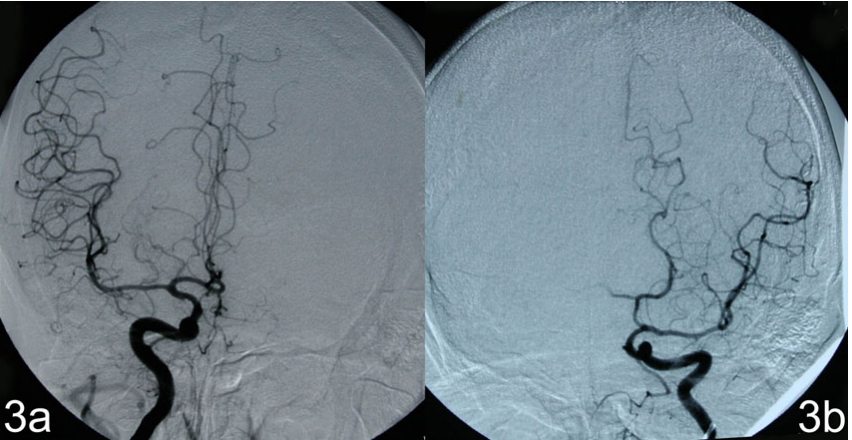
**Right and left internal carotid artery angiograms**. (A) Right internal carotid artery angiogram demonstrating the internal carotid artery dividing into middle and anterior cerebral arteries with the latter additionally supplying the left anterior cerebral artery territory. (B) Left internal carotid artery angiogram revealing the internal carotid artery branching into the middle carotid artery with absent contrast flow into the anterior cerebral artery territory. This may occur in vessel occlusion, but in this case it indicates an aplastic A1 segment.

On examination post-transfer he was found to be conscious, alert and orientated. The patient had a degree of receptive dysphasia, mild bilateral lower limb weakness and altered behaviour and personality, suggestive of frontal lobe disruption. MRI was reported as in keeping with the initial suggestion of a space-occupying lesion (Figure 2). Given the sudden onset of his symptoms, the possibility of vascular aetiology was considered. Digital subtraction angiogram (DSA) revealed a hypoplastic left anterior carotid artery with the left medial frontal territory being fed by branches of the contralateral pericallosal artery (an anatomical variation); see Figure 3. This finding reinforced the clinical suspicion of a bifrontal infarction and therefore no neurosurgical intervention was proposed.

CT and MRI repeated 2 weeks after the onset of symptoms showed interval reduction in oedema within what appeared to be subacute bilateral anterior cerebral artery (ACA) territory infarcts. The infarction was attributed to thrombo-embolism in the right ACA. Cranial CT carried out at the 12-month follow-up appointment revealed cortical volume atrophy consistent with a bifrontal parafalcine infarction. Eighteen months after his initial presentation, the patient has made a good recovery and is continuing cognitive rehabilitation therapy.

## Discussion

The ACA is a major vessel responsible for the blood supply to the interhemispheric region. Infarction of the ACA territory accounts for only 0.3% to 4.4% of cerebral infarctions reported [4,5]. Bilateral ACA infarction is even rarer. Twenty-seven cases of ACA territory infarction were reported among 1490 cases of cerebral infarction in the Lausanne Stroke Registry; however, there were only two cases of bilateral ACA territory infarction [6].

According to Bogousslavsky and Regli, 63% of ACA infarctions result from cardiogenic emboli or artery-to-artery emboli [6]. Gacs et al. reported other causes: unilateral occlusion of the internal carotid artery (ICA), distal extensions of ICA thrombosis and local thrombus caused by vasculitis [5]. Bilateral ACA territory infarction is usually due to vasospasm that occurs as a complication of subarachnoid haemorrhage caused by the rupture of one or more aneurysms of the anterior communicating arteries or distal ACAs [4]. However, in the case of an anomaly in the anterior part of the circle of Willis, thrombosis or embolism can lead to bilateral infarction.

Anomalies of the ACA are not quite as rare as was previously believed [7,8]. Baptista demonstrated anomalies of the ACA in 25% of the brain specimens studied [7]. Considerable variations occur in the origin and course of the ACA [8]. However, three distinct patterns are well recognised: accessory ACA, bihemispheric ACA and unpaired or azygous ACA [8]. The true incidence of bilateral ACA infarction is unknown, with few cases reported in the literature. In 2004, Yamaguchi et al. reported a similar case with a patient presenting with lower limb weakness and magnetic resonance angiography demonstrating bilateral anaplastic ACAs [9].

In our patient, DSA demonstrated a hypoplastic left ACA and branches of the right ACA supplying part of the left frontal lobe whilst serial MRI excluded the possibility of a neoplasm. Serial CT scans confirmed that this was indeed a case of infarction. If investigations were not performed to confirm or exclude the clinical suspicion of cerebrovascular infarction despite the findings of the initial cranial CT, a diagnosis of bilateral ACA territory infarction would have been delayed or missed and the patient might have had to undergo an unnecessary surgical intervention. However, it should be noted that other imaging, for example CT angiography or CT perfusion studies, if performed nearer the time of presentation could have resulted in an accurate diagnosis more acutely.

## Conclusion

This case report highlights the finding that simultaneous bilateral cerebral infarction can be the result of a unilateral cerebral artery occlusion and that this can potentially mimic a space-occupying lesion. It also demonstrates that anomalies of cerebral vasculature are not as rare as is usually believed, and this should be borne in mind when investigating unusual presentations of cerebrovascular infarction.

## Abbreviations

ACA: anterior cerebral artery; CT: computerised tomography; DSA: digital subtraction angiography; ICA: internal carotid artery; MRI: magnetic resonance imaging.

## Competing interests

The authors declare that they have no competing interests.

## Authors' contributions

BFM made substantial contributions to the design, acquisition of data, literature review and drafting of this manuscript, BC contributed to the acquisition of data and the radiological images, JK and DO were responsible for the conception, drafting and general supervision of this work. All authors have given final approval of the version to be published.

## Consent

Written informed consent was obtained from this patient for the publication of this case report and any accompanying images. A copy of the written consent is available for review by the Editor-in-Chief of this journal.

## References

[B1] Hodes PJ Campoy F Riggs HE Bly P Cerebral angiography: fundamentals in anatomy and physiology Am J Roentgenol Radium Ther Nucl Med 1953 70 61 82 13058026

[B2] Krabbe-Hartkamp MJ Grond J van der de Leeuw FE de Groot JC Algra A Breteler MM Mali WP Circle of Willis: morphologic variation on three-dimensional time-of-flight MR angiograms Radiology 1998 207 103 11 9530305 10.1148/radiology.207.1.9530305

[B3] Maurer J Maurer E Perneczky A Surgically verified variations in the A1 segment of the anterior cerebral artery J Neurosurg 1991 75 950 953 1823542 10.3171/jns.1991.75.6.0950

[B4] Orlandi G Moretti P Fioretti C Puglioli M Collavoli P Murri L Bilateral medial frontal infarction in a case of azygous anterior cerebral artery stenosis Ital J Neurol Sci 1998 19 106 108 10.1007/BF02427567 10935847

[B5] Gacs G Fox AF Barnett HJ Vinuela F Occurrence and mechanisms of occlusion of the anterior cerebral artery Stroke 1983 14 952 959 6659000 10.1161/01.str.14.6.952

[B6] Bogousslavsky J Regli F Anterior cerebral artery territory infarction in the Lausanne Stroke Registry. Clinical and etiologic patterns Arch Neurol 1990 47 144 150 2302085 10.1001/archneur.1990.00530020040012

[B7] Baptista AG Studies on the arteries of the brain. II. The anterior cerebral artery: some anatomic features and their clinical implications Neurology 1963 13 825 835 14066997 10.1212/wnl.13.10.825

[B8] Critchley M The anterior cerebral artery and its syndromes Brain 1930 53 120 165 10.1093/brain/53.2.120

[B9] Yamaguchi K Uchino A Sawada A Takase Y Kuroda Y Kudo S Bilateral anterior cerebral artery territory infarction associated with unilateral hypoplasia of the A1 segment: report of two cases Radiat Med 2004 22 422 425 15648459

